# Retroperitoneal haemorrhage in renal angiomyolipoma causing hepatic functional decompensation: a case report

**DOI:** 10.1186/1752-1947-1-82

**Published:** 2007-09-06

**Authors:** Julekha R Wajed, Simon D Taylor-Robinson, James E Jackson, Gordon WH Stamp

**Affiliations:** 1 Divisions of Medicine, Imperial College London, Hammersmith Hospital Campus, Du Cane Road, London W12 0NN, UK; 2 Imaging Sciences, Imperial College London, Hammersmith Hospital Campus, Du Cane Road, London W12 0NN, UK; 3 Investigative Sciences, Imperial College London, Hammersmith Hospital Campus, Du Cane Road, London W12 0NN, UK

## Abstract

Renal angiomyolipomata usually present as incidental findings on routine imaging, but rarely they may give rise to significant haemorrhage. If bleeding occurs, first-line treatment is currently angiography with selective embolisation. Prophylactic embolisation may be considered in some cases, depending on lesion size and patient co-morbidities.

We present a case of retroperitoneal bleeding from a renal angiomyolipoma in a patient with known cirrhosis of the liver, which caused acute deterioration of liver function and consequent hepatic encephalopathy. Selective embolisation of the lesion was performed with a good subsequent outcome. Such functional hepatic decompensation has not previously been reported in this context and we suggest the use of prophylactic embolisation for incidental renal angiomyolipomata, regardless of size, in all patients with chronic liver disease to prevent this potentially life-threatening complication.

## Background

Renal angiomyolipomata are incidental findings that usually remain clinically silent in the majority of cases [1]. However, in a small minority there have been reports of severe haemorrhage, which can be fatal if not treated promptly [2-6].

There is a strong association with angiomyolipomata and tuberous sclerosis [2,4,7]. However, there are no case reports of renal angiomyolipomata in patients with chronic liver disease and the potential hepatic complications, if there is a spontaneous haemorrhage [8-10].

There is an established risk of sudden bleeding from angiomyolipomata in lesions greater than 4 cm in maximal diameter, or during pregnancy from an increased haemodynamic flow. In some countries this has led to prophylactic embolisation beginning to become the recommended practice in such cases [4-6,11].

This case report demonstrates the rare complication of a bleeding renal angiomyolipoma in association with chronic liver disease, which caused rapid decompensation and encephalopathy. We suggest the use of prophylactic embolisation of incidental angiomyolipomata, regardless of size, in all patients with chronic liver disease.

## Case history

A 59 year-old woman presented to her general practitioner in 2005 with a 2-month history of right-sided flank pain, haematuria, and weight loss. She was not diabetic or dyslipidaemic, and there was no significant past medical or family history. She did not take any medications and denied any significant alcohol consumption, cigarette smoking or a history of illicit intravenous drug usage.

Examination revealed hepatomegaly with tenderness in the right upper quadrant. There was no evidence of jaundice, ascites or hepatic encephalopathy and rest of system examinations were normal.

Initial blood tests revealed abnormal liver function tests (LFTs), with alanine aminotransferase (ALT) 38 u/L (normal range: 5–40 u/L), alkaline phosphatase (ALP) 142 u/L (normal range: 20–120 u/L), bilirubin 35 mmol/L (normal range < 17 mmol/L), and gamma glutamyl transferase (GGT) 335 (normal range: < 55 u/L). Her prothrombin time was not significantly prolonged at 12.1 s (normal range 9.6–11.6 s).

In view of the above, she was referred to our hospital for further investigations. Additional blood tests included an autoimmune antibody screen, serum copper, caeruloplasmin, alpha-1- antitrypsin and serum ferritin levels, which were all normal. Hepatitis virology and serum tumour markers were also all negative.

**Figure 1:**
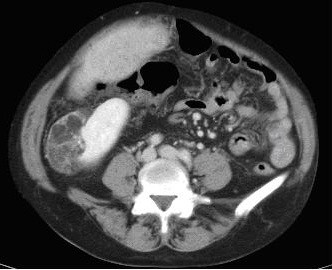
Axial CT image through the abdomen demonstrates a well-defined mass arising from the lower pole of the right kidney containing fat and soft tissue elements consistent with an angiomyolipoma.

Radiological examination included an abdominal ultrasound scan (USS), which showed hepatomegaly with dilatation of the pancreatic and common bile ducts. Computed tomography (CT) of the abdomen revealed an irregularly enlarged liver of heterogeneous attenuation, with a nodular right lobe in keeping with cirrhosis. A 5 cm × 4 cm lesion was also noted in the lower pole of the right kidney that consisted of fat and soft tissue elements, characteristic of a renal angiomyolipoma (figure 1).

Magnetic resonance (MR) of the abdomen confirmed an enlarged liver with hypertrophy of the caudate lobe and numerous focal nodules, likely to represent regenerative nodules. There was also evidence of chronic pancreatitis, and the appearance of a right-sided renal angiomyolipoma was confirmed.

An ultrasound-guided liver biopsy was performed. Histology from this revealed appearances of cirrhosis, although no specific underlying cause was evident. As a result, she was diagnosed as having compensated 'cryptogenic' cirrhosis of the liver, with chronic pancreatitis and a right-sided renal angiomyolipoma.

A year later, she was admitted through the accident and emergency department with vomiting, fever and severe back pain, radiating down both legs. She denied any passage of melaena, rectal bleeding or haematemesis and there were no obvious precipitating causes for her symptoms.

On examination she appeared pale, jaundiced and there was a marked postural drop in her blood pressure. She was tender in the right upper abdominal quadrant and right loin, with hepatomegaly and gross ascites.

Blood tests revealed a 3 g/dL drop in haemoglobin to 8.6 g/dL and worsening LFTs (ALT 55 u/L, bilirubin 48 mmol/L and GGT 478 u/L). Arterial blood gases showed a high anion gap with metabolic acidosis and respiratory compensation. Her prothrombin time was slightly elevated at 13.1 s with an APTT at the upper end of the normal range at 31.8s (normal range 24–32 s) and a normal fibrinogen level at 2.53 g/L (normal range 1.8–3.6 g/L).

**Figure 2:**
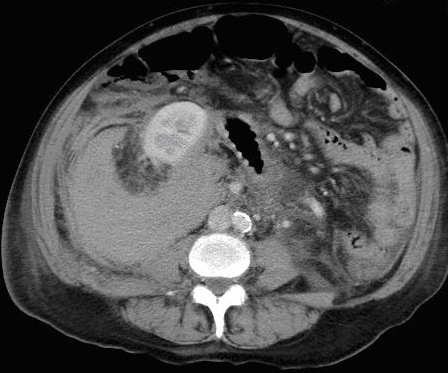
Axial CT image demonstrates a large right retroperitoneal haematoma surrounding the angiomyolipoma and displacing the right kidney anteriorly.

Resuscitation with intravenous fluids and blood products took place immediately. When stable, an urgent oesophageo-gastro-duodendoscopy (OGD) was performed. This did not localise any ulcers or bleeding points. There was no evidence of altered blood in the stomach. However, an abdominal CT scan revealed an 8 cm diameter haematoma in the retroperitoneal space, adjacent to and displacing the right kidney (figure 2). The findings were consistent with an acute haemorrhage from the incidental renal angiomyolipoma found on previous scans.

Unfortunately, her clinical condition deteriorated very quickly. She became confused, encephalopathic, and her Glasgow coma scale (GCS) dropped to 11/15. The patient was transferred to the intensive care unit, where she was intubated and ventilated. She was transfused with blood products and intravenous cefotaxime and metronidazole were given to avoid the risk of spontaneous bacterial peritonitis.

**Figure 3:**
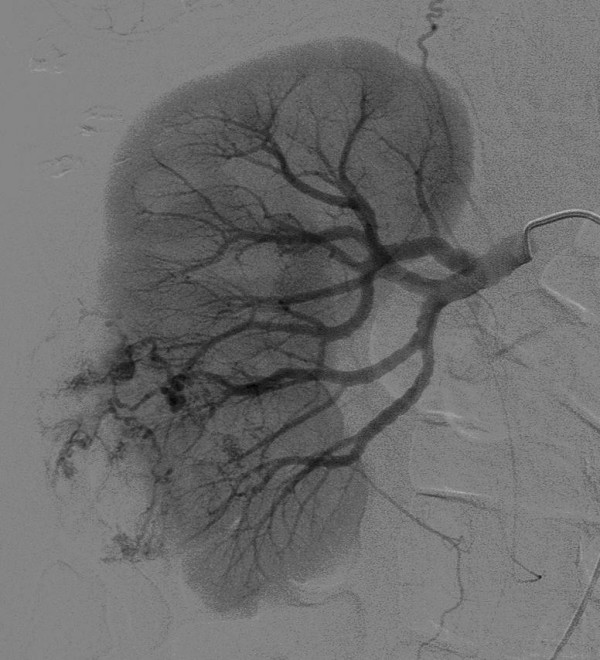
Selective right renal artery angiogram demonstrates abnormal vessels containing areas of aneurysmal dilatation supplying the lower pole angiomyolipoma.

In view of the CT scan results, a renal angiogram was performed when she became more haemodynamically stable. This highlighted the renal angiomyolipoma as a 4 cm diameter vascular mass arising from the lower pole of the right kidney. Its blood supply was derived from two separate arterial branches, one from the anterior division of the renal artery, and the other from the posterior division (figure 3).

**Figure 4:**
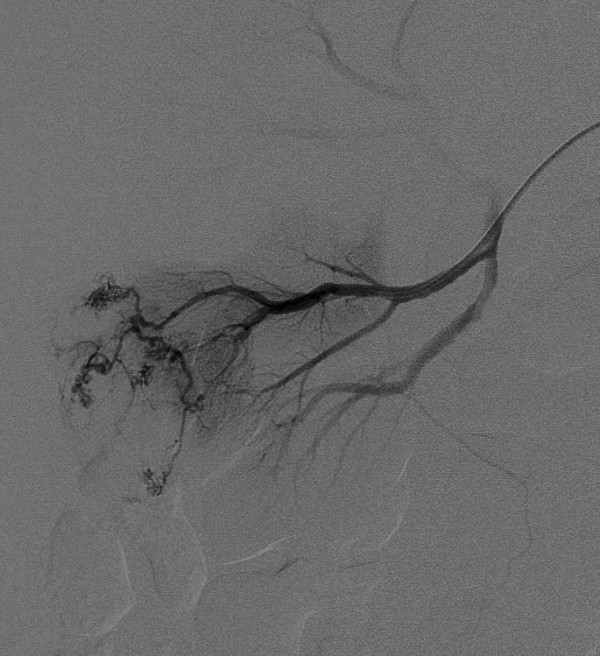
Selective right renal arterial branch angiogram demonstrates one of the feeding vessels to the tumour before embolisation.

Areas of arterial aneurysmal dilatation were present within the tumour vessels and a focal pseudoaneurysm was demonstrated arising from one of these abnormal arteries, from which active contrast medium extravasation was seen during subsequent selective catheterisation (figure 4).

**Figure 5:**
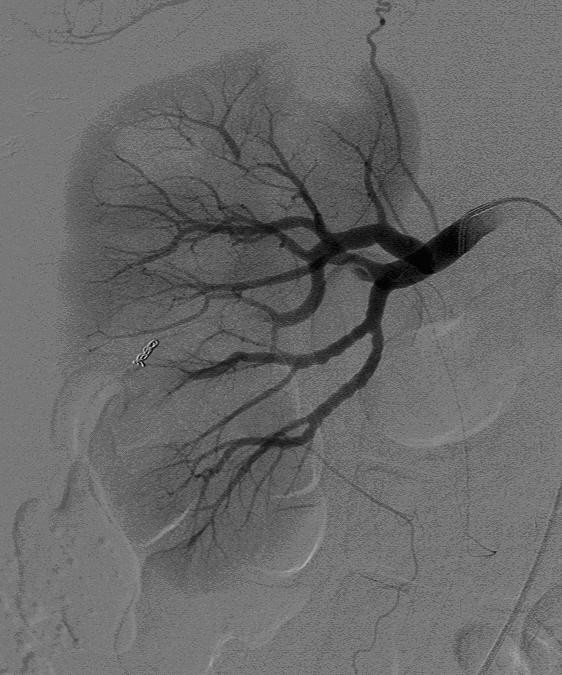
Selective right renal artery angiogram after embolisation demonstrates obliteration of the tumour supply.

Both feeding arteries were selectively catheterised and embolised with polyvinyl alcohol. After this, platinum microcoils were placed at the origin of the more diseased vessel that supplied the pseudoaneurysm (figure 5).

The patient remained haemodynamically stable after the procedure, and could be transferred out of intensive care. However, the recovery from her encephalopathy was much slower, taking around 10 weeks for her indices of hepatic synthetic function to return back to normal.

Treatment for her encephalopathy included L-ornithine L-aspartate, regular human albumin solutions, and bowel cleansing preparations that included lactulose and phosphate enemas. She was discharged 3 months after her initial presentation and remains well.

## Discussion

Renal angiomyolipomata arise from the mesenchymal elements of the kidney^2^. These tumours consist of a collection of blood vessels, smooth muscle and mature adipose tissue, although a more aggressive subtype also exists that consists of perivascular epitheliod cells [3,6,7,12]. 80% of angiomyolipomata are associated with tuberous sclerosis [2,6]. In this context, such lesions are typically multiple, bilateral and are associated with rapid growth and an increased risk of haemorrhage [4,7]. A sporadic and rare form of angiomyolipoma is also found where lesions are usually single, unilateral and generally asymptomatic [2,11].

Renal angiomyolipomata are commonly found as incidental findings on cross-sectional imaging (ultrasound, CT or MR) [1]. On ultrasound, they usually cast an acoustic shadow, and appear homogeneous with high reflectivity due to their high fat content [1,6]. On CT, angiomyolipomata have well-defined margins, with a variable proportion of fat and soft tissue, although the former usually predominates [3]. The fat content of these lesions is also well demonstrated on MR [1].

A fine-needle biopsy or aspiration cytology may be considered on rare occasions where the diagnosis may be in doubt, although there is a potential risk of serious haemorrhage during the procedure. Histology of angiomyolipomata confirms that they are mainly composed of spindle-shaped smooth muscle cells, and the presence of multiple epithelioid cells is found in the more aggressive form [3,12]. Genetic studies confirm a clonal neoplastic proliferation [2].

The majority of patients with renal angiomyolipomata are asymptomatic, and it is estimated that over 10 million people worldwide have such lesions [1]. In the minority of patients that are symptomatic, the classic 'Lenk's triad' of symptoms include flank or abdominal pain, a palpable or tender mass and haematuria^4^. Other symptoms may include fever, vomiting, anaemia, renal failure and hypotension [3].

Some angiomyolipomata can grow rapidly, up to 4 cm per year [13]. If greater than 4 cm in diameter, these lesions are associated with an increased risk of aneurysmal formation and hence a higher possibility of rupture and haemorrhage [2,4,11,13]. If bleeding presents acutely, it is potentially a life-threatening situation, which may require surgical intervention leading to a possible nephrectomy [7,11].

In all cases, the aim of treatment is to preserve renal function and to prevent haemorrhage [1]. The current first-line treatment is angiography with selective embolisation and all patients with symptoms should be considered for this intervention [2-4].

The role of prophylactic embolisation is to prevent the possible risk of haemorrhage [4]. It is currently recognized that in angiomyolipomata greater than 4 cm in diameter and the presence of intra-tumoral aneurysmal disease predisposes to an increased risk of bleeding. This is also true for women who intend to become pregnant, on account of haemodynamic changes during pregnancy [4-6,11].

Surgery is reserved for patients who have had an unsuccessful embolisation. It is also considered where there is a suspicion of malignancy, and in patients with persistent and life-threatening hemorrhages, which could possibly lead to a nephrectomy [4,7].

It is well recognised that the spectrum of hepatic encephalopathy (HE) varies from minimal encephalopathy, which can only be found by sensitive psychometric tests and imaging modalities, to overt HE, which can manifest as deep coma [8-10]. In the case we report, the patient had previously functioned normally with respect to her cognitive state. However, the episode of overt HE seemed to be caused by haemodynamic instability from the bleeding angiomyolipoma, which is a hitherto unreported risk of these renal lesions.

## Conclusion

We presented a rare case where an incidental 4 cm renal angiomyolipoma was found on routine screening, in a patient with known chronic liver disease. Spontaneous haemorrhage from this lesion caused sudden functional hepatic decompensation, which took over 10 weeks for a full recovery.

In view of the potential risks of bleeding and subsequent development of HE, the detection of a 4 cm angiomyolipoma on routine screening should have raised the possibility of prophylactic embolisation, especially in this patient with cirrhosis of the liver.

Although our patient did not have a significant coagulopathy on presentation, prolonged prothrombin times are a major feature of chronic liver disease, particularly during episodes of functional decompensation. This would make patients with coexisting angiomyolipomata more at risk of bleeding, than the general population. We therefore suggest the use of prophylactic embolisation of incidental angiomyolipomata if present, regardless of its size, in all patients with chronic liver disease.

## Competing interests

The author(s) declare that they have no competing interests.

## Authors' contributions

JRW, SDT-R and GWS looked after the patient. JEJ performed the renal embolisation. All authors contributed to the genesis and the writing of the manuscript. All authors have seen and approved the final document.
